# Growth Kinetics of Influenza C Virus Antigenic Mutants That Escaped from Anti-Hemagglutinin Esterase Monoclonal Antibodies and Viral Antigenic Changes Found in Field Isolates

**DOI:** 10.3390/v13030401

**Published:** 2021-03-03

**Authors:** Yoko Matsuzaki, Kanetsu Sugawara, Yoshitaka Shimotai, Yoko Kadowaki, Seiji Hongo, Katsumi Mizuta, Hidekazu Nishimura

**Affiliations:** 1 Department of Infectious Diseases, Yamagata University Faculty of Medicine, Yamagata 990-9585, Japan; ksugawar@med.id.yamagata-u.ac.jp (K.S.); yoshimo@med.id.yamagata-u.ac.jp (Y.S.); yokocadow@med.id.yamagata-u.ac.jp (Y.K.); shongou@med.id.yamagata-u.ac.jp (S.H.); 2 Department of Microbiology, Yamagata Prefectural Institute of Public Health, Yamagata 990-0031, Japan; mizutak@pref.yamagata.jp; 3 Virus Research Center, Clinical Research Division, Sendai Medical Center, Sendai 983-8520, Japan; hide-nishimura@mte.biglobe.ne.jp

**Keywords:** influenza C virus, escape mutant, antigenic drift, growth kinetics, surveillance

## Abstract

The antigenicity of the hemagglutinin esterase (HE) glycoprotein of influenza C virus is known to be stable; however, information about residues related to antigenic changes has not yet been fully acquired. Using selection with anti-HE monoclonal antibodies, we previously obtained some escape mutants and identified four antigenic sites, namely, A-1, A-2, A-3, and Y-1. To confirm whether the residues identified as the neutralizing epitope possibly relate to the antigenic drift, we analyzed the growth kinetics of these mutants. The results showed that some viruses with mutations in antigenic site A-1 were able to replicate to titers comparable to that of the wild-type, while others showed reduced titers. The mutants possessing substitutions in the A-2 or A-3 site replicated as efficiently as the wild-type virus. Although the mutant containing a deletion at positions 192 to 195 in the Y-1 site showed lower titers than the wild-type virus, it was confirmed that this region in the 190-loop on the top side of the HE protein is not essential for viral propagation. Then, we revealed that antigenic changes due to substitutions in the A-1, A-3, and/or Y-1 site had occurred in nature in Japan for the past 30 years. These results suggest that some residues (i.e., 125, 176, 192) in the A-1 site, residue 198 in the A-3 site, and residue 190 in the Y-1 site are likely to mediate antigenic drift while maintaining replicative ability.

## 1. Introduction

Influenza C virus was first isolated in 1947 (C/Taylor/1233/1947) from the throat washings of a man living in New York City who had a mild headache and backache and coryza [1]. Although influenza C virus can infect humans of all age groups [2,3], children account for the vast majority of clinical cases. This virus not only causes a mild upper respiratory tract illness in children but also can causes lower respiratory tract illness, such as bronchitis and pneumonia, particularly in children < 2 years old [2,4,5,6] or in patients with comorbidities [2].

Information about the antigenic characterization of influenza C virus is limited [7,8], because this virus is rarely isolated by cell culture due to technical difficulty. Since 1988, we have continuously monitored influenza C virus infections in Japan using a cell culture method and have carried out antigenic analysis of isolated strains. As a result, we revealed that influenza C viruses can be classified based on the hemagglutinin esterase (HE) gene sequence into six genetic and antigenic lineages, designated C/Taylor, C/Mississippi, C/Aichi, C/Yamagata, C/Kanagawa, and C/Sao Paulo [9]. Among the six HE lineages, five lineages, excepting C/Taylor, circulated in Japan between 1988 and 2018 [7,10,11,12,13,14,15,16,17,18,19]. Antibodies are frequently cross-reactive between lineages, yet differences in antibody reactivities can be observed [7]. Such stable antigenicity of the HE glycoprotein was also confirmed by a shared amino acid sequence identity of more than 94% for the HE proteins of natural isolates collected during a 68-year period [9]. As one of the reasons for the stable antigenicity, we previously proposed functional constraints on variation in the antigenic region of the HE glycoprotein [20], but no researchers have yet proved this hypothesis by experimental studies.

Influenza C virus is a member of the *Orthomyxoviridae* family of enveloped and segmented negative-sense RNA viruses, together with the influenza A, B, and D viruses. The HE protomer, which is encoded from the fourth segment of the viral genome, is composed of two subunits, HE1 (432 amino acids) and HE2 (209 amino acids) [21]. The HE glycoprotein forms trimers and carries major antigenicity determinants; it also exerts three biological activities: receptor-binding activity, fusion with the host cell membrane, and receptor-destroying activity [22,23,24]. Rosenthal et al. [25] determined three functional domains within the crystal structure of the HE protein: a receptor-binding domain comprising residues 151–310 and an esterase domain comprising residues 41–150 and residues 311–366 are the globular region of the HE protein, and a fusion domain comprising the remainder is the stalk region.

Recently, we mapped the neutralizing epitopes of influenza C virus onto the three-dimensional (3D) structure of the HE glycoprotein by using escape mutants selected by anti-HE monoclonal antibodies (MAbs) and identified four antigenic sites, namely, A-1, A-2, A-3, and Y-1 [26]. The A-1 site was widely located around the receptor-binding site, the A-2 site was near the receptor-destroying enzyme site, the A-3 site was located on the back side of the A-1 site, and the Y-1 site was located in the 190-loop on the top side of the HE protein.

The residues identified as the neutralizing epitope in these antigenic sites may possibly be associated with the antigenic drift of influenza C virus. To resolve the problem regarding the functional constraints on variation in the antigenic region, we examined the growth kinetics of these escape mutants and found some residues that affect the antigenicity while retaining replicative fitness. Then, we reviewed antigenic mutations that occurred in our surveillance work of the past 30 years and investigated the possibility of the occurrence of antigenic drift in nature.

## 2. Materials and Methods

### 2.1. Viruses

The escape mutants and their parental viruses were obtained from our previous study [26]. Ten mutants derived from C/Ann Arbor/1/50 possessing the R68W, L164P, N173I, N175S, S192L, E193K, K198E, K235R, D269N, or A351V substitution and two deletion mutants, namely ∆198 and ∆192–195, derived from C/Yamagata/15/2004, were used in this study. The influenza C viruses C/Yamagata/7/88, C/Yamagata/11/88, C/Yamagata/14/2004, and C/Yamagata/29/2004, which had been isolated in our previous studies [10,17], were all propagated in the amniotic cavities of 8- or 9-day-old embryonated hen eggs.

### 2.2. Hemagglutination Inhibition Test

Nine anti-HE MAbs (J9, U9, Q5, J14, K16, U1, U2, YA3, and YA5) characterized in our previous reports [26,27] were used for the antigenic analysis. The antigenic sites A-1, A-2, A-3, and Y-1 were identified by a competitive assay with antibodies and operational analysis with escape mutants [26,27]. The MAbs J9, U9, Q5, and J14 interact with the A-1 site; MAb K16 interacts with the A-2 site; MAbs U1 and U2 interact with the A-3 site; and MAbs YA3 and YA5 interact with the Y-1 site. Chicken antisera against C/Ann Arbor/1/50 (C/Taylor lineage) and C/Yamagata/10/89 (C/Yamagata lineage) were used as we previously described [7,28]. Hemagglutination inhibition (HI) tests were performed in 96-well microtiter plates. Briefly, 50 µL of 8 hemagglutinating units of virus was added to each well containing 50 µL of twofold serially diluted MAbs or antiserum. After incubation for 30 min at room temperature, 100 µL of 0.5% chicken erythrocytes was added to all wells, and the plates were stored for 60 min at 4 °C. The HI titer was expressed as the reciprocal of the highest antibody dilution, which completely inhibited hemagglutination. The HI test results of 509 strains isolated in our surveillance work were obtained from our previous experimental data.

### 2.3. Nucleotide Sequencing

Sequencing of seven gene segments (PB2, PB1, P3, HE, NP, M, and NS genes) for C/Yamagata/11/88 and C/Yamagata/29/2004 was performed as previously described [9]. Briefly, viral RNA was extracted from 140 μL of amniotic fluid using the QIAamp Viral RNA Mini kit (Qiagen, Hilden, Germany) and transcribed into complementary DNA (cDNA) using a universal primer (5′-AGCAGAAGCAGG-3′) that is complementary to positions 1–12 at the 3′-end of all influenza C virus RNA segments. Using the synthesized cDNA as a template, the complete coding regions of the individual RNA segments were amplified using gene-specific primers. The PCR products were purified using a QIAquick PCR purification kit (Qiagen, Hilden, Germany) and sequenced using a BigDye Terminator v3.1 cycle sequencing kit (Life Technologies, Carlsbad, CA, USA) and an ABI Prism 3130 sequencer (Applied Biosystems, Foster City, CA, USA). The primer sequences were reported in our previous paper [9]. The GenBank accession numbers for nucleotide sequences determined in this study are LC603641 to LC603654.

### 2.4. Structural Analysis

Amino acid positions were plotted on the 3D structure of HE molecules using the PyMOL Molecular Graphics System, version 1.8.6.1 (Schrödinger, LLC). The crystal structure of HE was obtained from the Protein Data Bank (PDB ID: 1FLC).

### 2.5. Viral Growth Kinetics

Madin-Darby canine kidney (MDCK) cells were infected with the indicated viruses at a multiplicity of infection (MOI) of 0.001 in the presence of 20 μg/mL TPCK-trypsin at 34 °C. The culture medium supernatants were harvested every 24 h from 1 to 6 days post-infection (dpi). Viral titers were determined by plaque assays with MDCK cells as described previously [29]. Briefly, serially 10-fold diluted supernatants were inoculated onto MDCK cell monolayers cultured in a 6-well plate. After incubation at 34 °C for 60 min, the cells were overlaid with Eagle’s minimum essential medium (MEM) containing 1% Avicel and 5 μg/mL TPCK-trypsin and incubated at 34 °C. At 4 dpi, the cells were fixed with 4% paraformaldehyde for 30 min at 4 °C, followed by incubation in 0.5% Triton-X-100 and 20 mM glycine in PBS for 15 min at room temperature. The cells were then reacted with anti-NP MAb H27 [30] as a primary antibody and goat antimouse IgG conjugated with horseradish peroxidase as a secondary antibody, and the plaque numbers visualized with TrueBlue (SeraCare, Milford, MA, USA) were counted. Experiments were performed in triplicate, and the values are expressed as the mean ± standard deviation (SD).

## 3. Results

### 3.1. Antigenic Properties of 12 Escape Mutants

We previously reported that some escape mutants of C/Ann Arbor/1/50 (C/Taylor lineage) and C/Yamagata/15/2004 (C/Yamagata lineage) have reduced HI activity against the MAbs used for selection [26]. To further evaluate the antigenic properties of these mutants, we performed an HI assay with seven MAbs against C/Ann Arbor/1/50 (J9, U9, Q5, J14, K16, U1, and U2), two MAbs against C/Yamagata/15/2004 (YA3 and YA5), and two polyclonal chicken antisera against C/Ann Arbor/1/50 and C/Yamagata/10/89 (C/Yamagata lineage) (Table 1 and Table 2). All twelve mutants analyzed greatly reduced the HI activity against the selected MAbs, and some mutants changed the reactivities against the MAbs belonging to the different antigenic sites. Although wild-type C/Ann Arbor/1/50 cannot react with MAbs YA3 and YA5 (Y-1 site), the mutant containing S192L selected by MAb U9 (A-1 site) and the mutant containing L164P selected by MAbs U1 and U2 (A-3 site) showed increased reactivity with MAbs YA3 and YA5. The deletion mutants Δ192–195 selected by MAbs YA3 and YA5 failed to react with not only the selected MAbs but also the MAb U9 belonging to the A-1 site, and another deletion mutant Δ198 selected by MAb YA3 showed loss and decreased reactivities with MAb U9 and Q5, respectively. These results indicate that epitopes YA3 and YA5 overlapped with epitope U9 and perhaps with epitopes U1 and U2.

We tested the antigenic cross-reactivity of the mutants with chicken antisera raised against viruses from the C/Taylor and C/Yamagata lineages. Although antigenic differences between mutants of C/Ann Arbor/1/50 and mutants of C/Yamagata/15/2004 were detected using polyclonal antisera, all mutants reacted with both antisera. Among them, we found that a mutant carrying the substitutions E193K or D269K had a two- or four-fold decrease in cross-reactivity, compared to the wild-type C/Ann Arbor/1/50, and the Δ198 deletion mutant virus had a four- or eight-fold decrease in cross-reactivity, compared to the wild-type C/Yamagata/15/2004.

### 3.2. Growth Kinetics of the Escape Mutants in Cultured Cells

**Figure 1:**
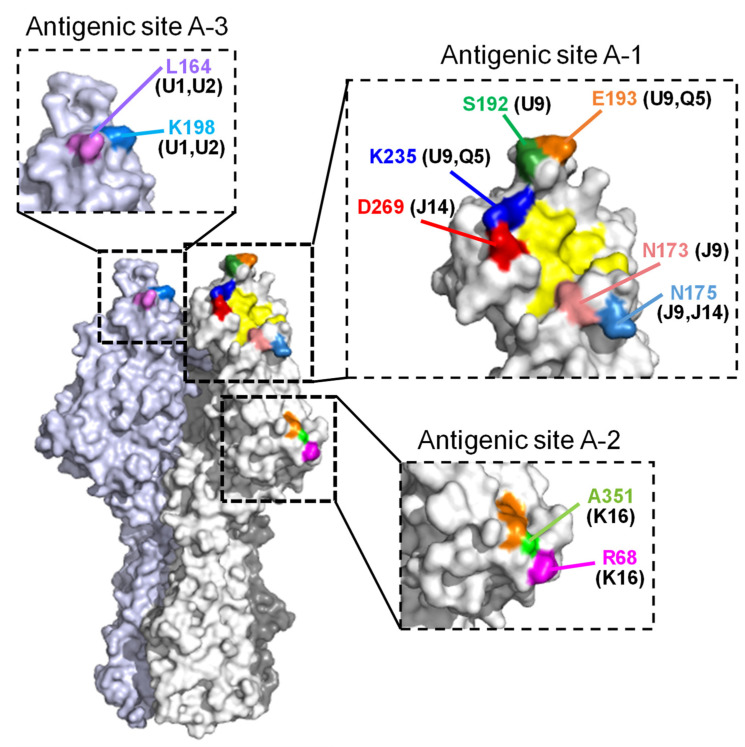
Positions of amino acid changes in the escape mutants are located on the hemagglutinin esterase (HE) molecule. Amino acid positions are designated by numbering, excluding the 14-amino-acid-long signal peptide. The MAbs used for selection are presented in parentheses. A trimer complex, in which monomers are white, gray, and light blue, is shown in surface representation. The residues involved in receptor binding and receptor destruction are colored yellow and orange, respectively.

To examine the replicative fitness of the viruses possessing mutations in the antigenic sites (Figure 1), we compared the growth kinetics of the escape mutant viruses in cultured cells with those of their wild-type virus. Using the 12 escape mutants listed in Table 1 and Table 2, we performed growth assays in MDCK cells. Daily virus titers from 1 to 6 dpi were determined by plaque assays with MDCK cells.

**Figure 2:**
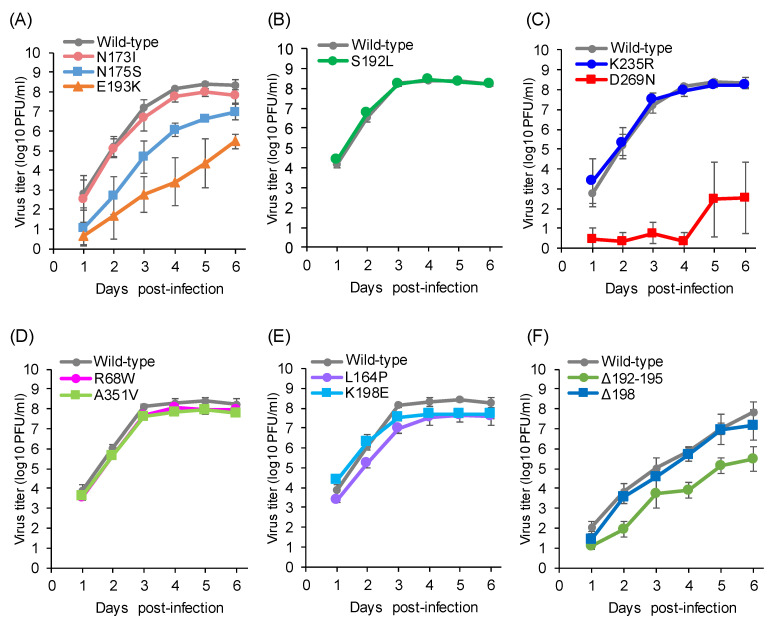
Replication kinetics of the escape mutants possessing substitutions in the A-1 site (**A**–**C**), A-2 site (**D**)**,** A-3 site (**E**), and Y-1 site (**F**). The growth kinetics of the wild-type C/Ann Arbor/1/50 (**A**–**E**) or the wild-type C/Yamagata/15/2004 (**F**) and the indicated mutant viruses in MDCK cells were compared. MDCK cells infected at a multiplicity of infection (MOI) of 0.001. Viral titers are presented as the mean ± standard deviation (SD) of three independent experiments.

Some viruses with mutations in antigenic site A-1, including the N173I, S192L, and K235R mutations, were able to replicate to titers comparable to that of the wild-type C/Ann Arbor/1/50 (Figure 2A–C), while others showed reduced titers. The N175S mutant virus, which acquires a new glycosylation site at position 173 instead of position 175, showed lower titers from 1 to 5 dpi (Figure 2A). The E193K or D269N mutant viruses, which had a two- or four-fold decrease in HI activity with antiserum raised against C/Ann Arbor/1/50, showed lower titers, compared with the wild-type C/Ann Arbor/1/50, from 1 to 6 dpi (Figure 2A,C).

The escape mutants possessing the R68W or A351V mutation in the A-2 site replicated as efficiently as the wild-type C/Ann Arbor/1/50 (Figure 2D). For the A-3 site, the L164P or K198E mutant virus showed similar virus titers, compared with the wild-type C/Ann Arbor/1/50 (Figure 2E). These results suggest that these mutants escaped neutralizing antibodies while maintaining replicative ability.

For the Y-1 site, the Δ198 deletion mutant virus replicated as efficiently as the wild-type C/Yamagata/15/2004, while the Δ192–195 deletion mutant showed lower titers from 2 to 6 dpi (Figure 2F). Nevertheless, the latter result suggests that deletions within the 190-loop are not critical for virus propagation.

### 3.3. Antigenic Changes among the Natural Isolates of Influenza C Virus in Japan between 1988 and 2018

**Figure 3:**
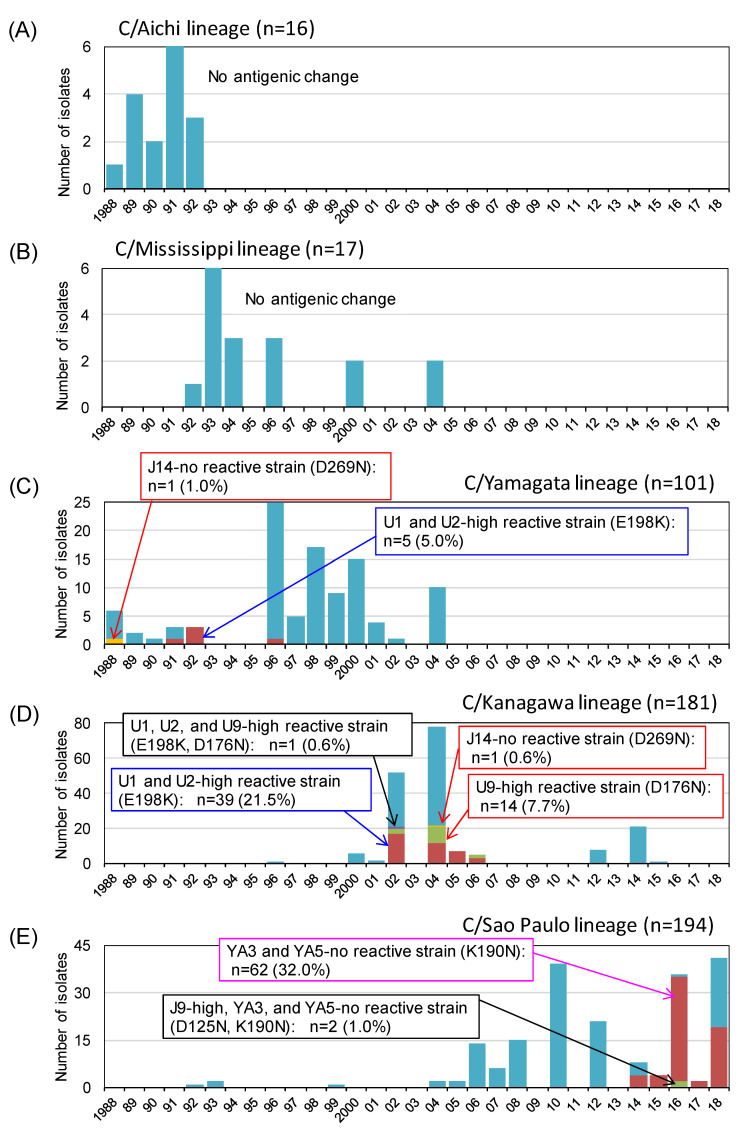
Antigenic changes among the natural isolates of influenza C virus in Japan between 1988 and 2018. Yearly number of isolates belonging to the five HE lineages and the frequency of antigenically different isolates within each of lineages are shown based on our previous reports [7,10,11,12,13,14,15,16,17,18,19,31]. Antigenic changes in the A-1 site, A-3 site, and Y-1 site are indicated as red, blue, and magenta squares, respectively. (**A**) C/Aichi lineage; (**B**) C/Mississippi lineage; (**C**) C/Yamagata/lineage; (**D**) C/Kanagawa lineage; and (**E**) C/Sao Paulo lineage.

Since 1988, we generated 509 HI test results for the antigenic analysis of influenza C virus isolates in Japan [7,10,11,12,13,14,15,16,17,18,19,31]. The yearly number of isolates belonging to the five HE lineages and the frequency of antigenically different isolates within each lineage are presented in Figure 3. 

**Figure 4:**
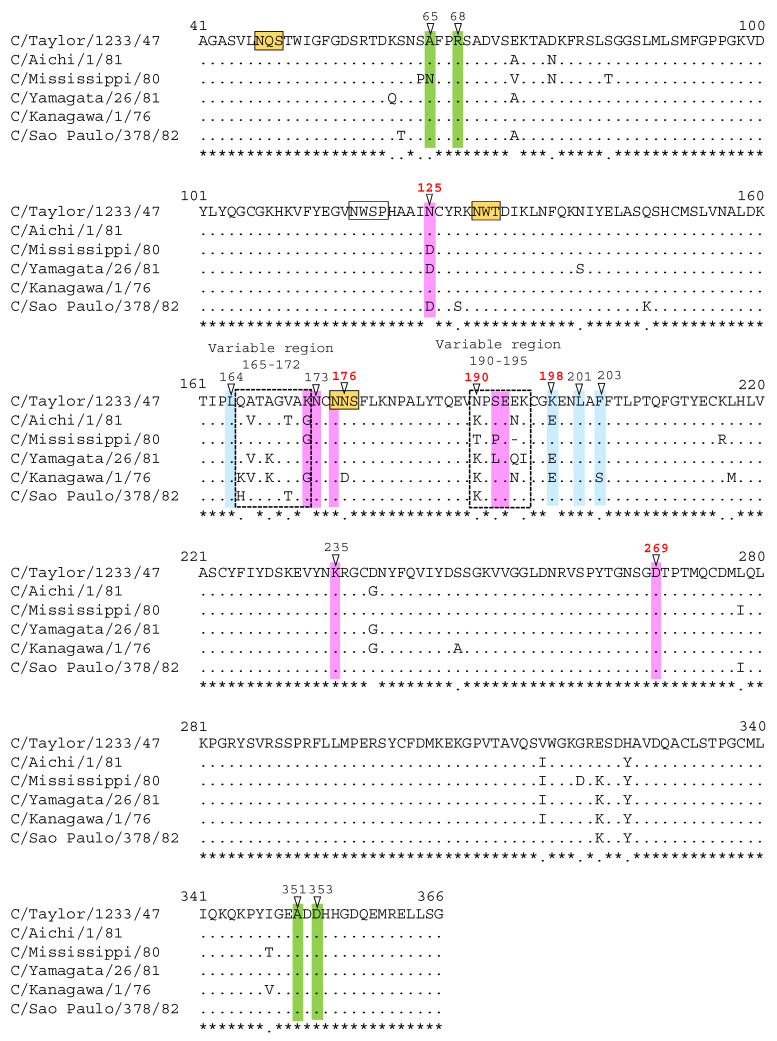
Amino acid sequence alignment of the HE globular region (amino acid positions from 41 to 366) of the reference strains belonging to the six HE lineages. The positions of amino acid substitutions that alter antigenicity in natural isolates are indicated by red letters. Colored residues indicate the antigenic sites A-1 (pink), A-2 (green), and A-3 (blue). Boxed residues colored yellow indicate the potential N-glycosylation sites, and noncolored boxed residues indicate a nonutilized sequon NWSP [25,32,33].

HI titers and amino acid sequence alignment of the reference strains of six HE lineages are shown in supplementary Table S1 and Figure 4, respectively. 

**Figure 5:**
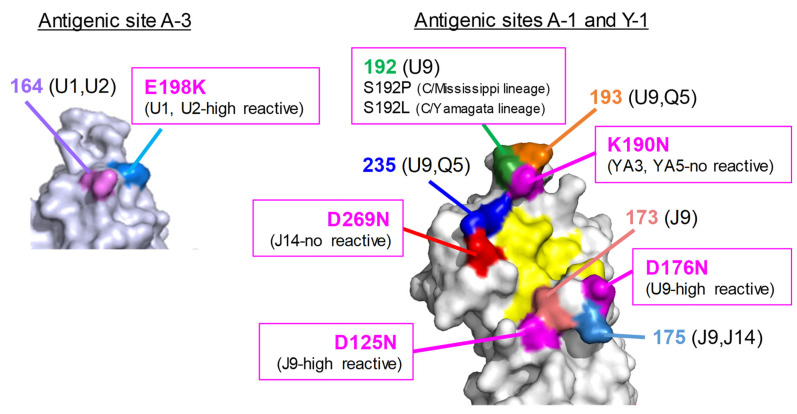
Positions of amino acid changes in the escape mutants and natural isolates. The amino acid changes that occurred in natural isolates are colored magenta. The residues involved in receptor binding are colored yellow.

The locations of the amino acid changes that occurred in natural isolates are shown in Figure 5, and the HI titers of the representative antigenic mutants are shown in supplementary Table S2. 

As shown in Figure 3, no antigenic change was detected in the HI tests among the strains belonging to the C/Aichi lineage or C/Mississippi lineage. For the C/Yamagata lineage, we isolated a J14-nonreactive strain possessing the D269N mutation (A-1 site) in 1988 (C/Yamagata/11/88), and five strains possessing the E198K mutation (A-3 site) showed 100-fold higher reactivity to the U1 and U2 MAbs than to the C/Yamagata lineage reference strain. However, these mutations were not inherited in further strains after 1996. We detected a large outbreak of C/Kanagawa lineage strains twice in 2002 and 2004 and found more than 100-fold higher reactivities to MAb U9 and MAbs U1 and U2 in 7.7% and 21.5% of these lineage strains, possibly due to the D176N (A-1 site) and E198K (A-3 site) mutations, respectively [31]. Moreover, only one strain possessing the D269N mutation (A-1 site), which failed to react with MAb J14, was isolated in 2004 (C/Yamagata/29/2004) for the C/Kanagawa lineage. 

In Japan, the isolation of C/Sao Paulo lineage strains has increased since 2006, and antigenic variants possessing the K190N mutation (Y-1 site), which failed to react with MAbs YA3 and YA5, have increased since 2014 and accounted for 32% of total strains by 2018. Among them, two strains isolated in 2016 showed a 100-fold higher reactivity to J9 due to the further mutation at position 125 (D125N) (A-1 site) [19].

**Figure 6:**
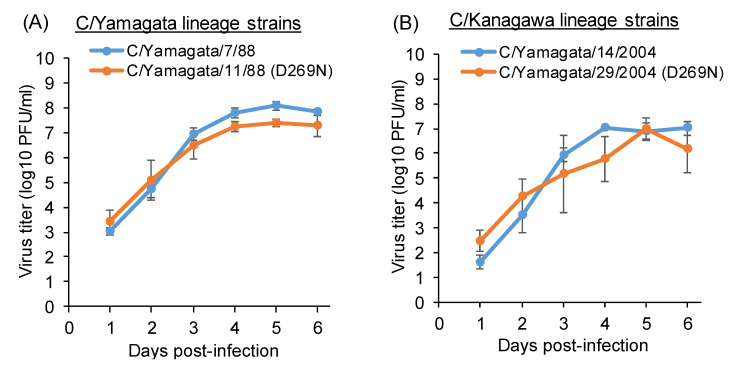
Replication kinetics of natural isolates with or without the D269N mutation among the C/Yamagata lineage strains (**A**) and C/Kanagawa lineage strains (**B**). Viral titers are presented as the mean ± SD of three independent experiments.

Since the escape mutant virus with the D269N mutation (A-1 site) replicated to lower titers than the wild-type C/Ann Arbor/1/50 (Figure 2C), we examined the growth kinetics of the C/Yamagata/11/88 and C/Yamagata/29/2004 strains possessing the D269N mutation and compared them with those of contemporary strains belonging to the same lineage, namely, C/Yamagata/7/88 and C/Yamagata/14/2004, respectively (Figure 6). When the whole genome sequences were compared, there was another amino acid difference in the stalk region of the HE protein (N505D) between C/Yamagata/7/88 and C/Yamagata/11/88, and there was only one amino acid difference in the M1 protein (K183N) between C/Yamagata/14/2004 and C/Yamagata/29/2004. As shown in Figure 6, the C/Yamagata/11/88 and C/Yamagata/29/2004 strains replicated to titers comparable to those of the respective strains without the D269N mutation.

## 4. Discussion

Evolutionary tree analyses have revealed that six antigenic lineages of influenza C virus diverged by approximately 1980 [5,34]. Although the C/Sao Paulo lineage and the C/Kanagawa lineage are now circulating worldwide [2,3,18,19,35,36], antigenic cross-reactivity between the C/Ann Arbor/1/50 (C/Taylor lineage) and recent isolates in Japan has been still confirmed [19]. We investigated whether the stable antigenicity of influenza C virus relates to the functional constraints on variation in the antigenic region of the HE glycoprotein.

We revealed that two variable regions, residues 165 to 172 and residues 190 to 195, exist in the A-1 site of the HE molecule, suggesting that six different antigenic lineages were generated by the stepwise accumulation of mutations that occurred in these regions (Figure 4). The A-1 antigenic site surrounds the receptor-binding site (Figure 1). One variable region including residues 165 to 172 is located on the left edge of the receptor-binding site, and another region including residues 190 to 195 is located in the 190-loop on the top side of the HE protein. Among the escape mutants possessing the amino acid change in the A-1 site, viruses with substitutions at residues 173, 192, and 235 were able to replicate to titers comparable to those of wild-type virus. Although each mutation at residues 173 and 235 was not observed among the natural isolates, S192P and S192L substitutions were found in viruses belonging to the C/Mississippi lineage and the C/Yamagata lineage, respectively (Figure 4 and Figure 5), suggesting that an amino acid change at position 192 is responsible for antigenic variation among the lineages.

In the A-1 site, the two escape mutants possessing the E193K or D269N mutation have affected their replicative fitness. Although amino acid differences at each position were not found between lineages (Figure 4), a strain possessing the D269N mutation was found in the C/Yamagata lineage (C/Yamagata/11/88) and in the C/Kanagawa lineage (C/Yamagata/29/2004). Interestingly, both strains replicated as efficiently as the respective reference strain without the D269N mutation, suggesting that the affected biological activity observed for the escape mutant with the D269N mutation was recovered in natural isolates by other amino acid variations; therefore, such strains may have acquired the possibility of survival in nature. However, the D269N mutation was not inherited in further strains of either the C/Yamagata lineage or the C/Kanagawa lineage, and the decreased reactivity with the polyclonal antibodies observed in the escape mutant possessing the D269N mutation (Table 1) was not found clearly in the C/Yamagata lineage (supplementary Table S2). Such a phenomenon was also observed in natural isolates possessing the E193K mutation: decreased reactivity with the polyclonal antibodies observed for the escape mutant possessing the E193K mutation (Table 1) disappeared in a natural isolate of the C/Taylor lineage (C/Paris/1/67), which possesses lysine at position 193 [26]. When these results are considered, it is unlikely that an amino acid change at position 193 or 269 contributes to generating an antigenic drift strain.

For the A-2 site, the MAb K16, which recognizes near the 9-O-acetylesterase active site [26], had little HI activity. Therefore, we could not show antigenic changes in natural isolates by HI assay using the MAb K16. In this study, however, we revealed that residues of the K16 epitope were able to change while maintaining the replicative ability. We previously reported that amino acid differences between two sublineages of the C/Sao Paulo lineage concentrated on the esterase domain of the HE protein [19]. Further analysis is needed to reveal whether the neutralization epitope of the C/Sao Paulo lineage exists in the esterase domain.

In the A-3 site, escape mutants possessing the L164P or K198E mutation replicated as efficiently as the wild-type virus. Although variation at residue 164 was not observed among the natural isolates, the K198E substitution was found in viruses belonging to the C/Aichi, C/Yamagata, and C/Kanagawa lineages (Figure 4). Some strains within the C/Yamagata lineage or C/Kanagawa lineage acquired the E198K mutation during the epidemic period, resulting in recovery of high reactivity with MAbs U1 and U2. Moreover, the Δ198 deletion mutant of the C/Yamagata lineage showed a four- to eight-fold decrease in reactivity with polyclonal immune sera while maintaining replicative ability. Therefore, an amino acid substitution at position 198 is likely to mediate antigenic drift in nature.

It is well known that the glycosylation of HA on influenza A viruses interferes with antibody access [37,38]. However, the number of N-glycosylation sequons (NXS/T, where X is any amino acid except P) in the HE protein did not increase at all for any lineage of influenza C virus [31]. As shown in Figure 4, three conserved glycosylation sites, at positions 47, 130 and 175, are utilized in the globular region of the HE protein [25,32,33]. Although the K190N mutation (Y-1 site), which was found in isolates of the C/Sao Paulo lineage after 2014, fit NXS/T motif at positions 190 to 192, a new glycosylation site is not created due to a proline at position 191, similar to the C/Taylor lineage (Figure 4). Therefore, antigenic changes occurred in the C/Sao Paulo lineage with the K190N mutation depending on an amino acid substitution at position 190, not on additional glycosylation. We have reported for the first time that antigenic variants with K190N mutations have spread worldwide as well as in Japan [19]. On the other hand, antigenic changes occurred in the C/Kanagawa lineage, which increased the reactivity of the MAb U9 and may be related to glycosylation at position 175 (A-1 site). The MAb U9-highly reactive strains of the C/Kanagawa lineage possessed the D176N substitution, but residue 176 was far from epitope U9 (positions 192 and 193 [26]); therefore, the oligosaccharide chain at position 175 may have influenced the reactivity with epitope U9 in the 190-loop on the top side of the HE protein. Furthermore, we revealed that the N175S mutant virus, which acquires a glycosylation site at position 173 instead of position 175, slightly affected the replicative ability. Despite this disadvantage, the N175S mutation may have a comparative advantage in mediating antigenic drift, since glycosylation at position 173 could alter the antigenicity of both epitope J9 (positions 125, 172, 173, and 175 [26]) and epitope J14 (mainly position 269 [26]).

For the Y-1 site, we examined the growth kinetics of the deletion mutant at positions 192 to 195, which failed to react with MAb U9 (A-1 site) and MAbs YA3 and YA5 (Y-1 site), and confirmed that this region in the 190-loop on the top side of the HE protein is not essential for viral propagation. This region is also variable among the lineages; therefore, further accumulation of mutations occurring in this region would contribute to antigenic changes within the lineage, such as the C/Sao Paulo lineage [19], and might become a sign of the generation of a new antigenic lineage.

Finally, although antigenic drift may be a rare or nonoccurring event for the influenza C virus, our long-term surveillance work indicated that the replacement of the dominant antigenic lineage periodically occurs [7]. We suggested that such replacement of the dominant lineage may be caused by immune selection within older children and/or adults who have pre-existing antibodies [7] and recently confirmed serologically that influenza C virus infections in the adult population had occurred in the epidemic period in children [39]. Therefore, there is always a possibility that an antigenic drift strain, including a new antigenic lineage, emerges, and further surveillance studies are necessary.

## 5. Conclusions

We revealed that some residues (i.e., 125, 176, 192) in the A-1 site, residue 198 in the A-3 site, and residue 190 in the Y-1 site are likely to mediate antigenic drift while maintaining replicative ability, and some residues (i.e., 173, 193, 235) in the A-1 site and residue 164 in the A-3 site are unlikely to be involved in antigenic drift. These findings may facilitate future surveillance of influenza C virus. 

## Figures and Tables

**Table 1:** Antigenic properties of escape mutants of the C/Ann Arbor/1/50 virus in the hemagglutination inhibition (HI) assay.

Viruses	Amino Acid Substitution	HI Titer of MAbs ^1^									HI Titer of Chicken Antiserum against	
		A-1 Site				A-2 Site	A-3 Site		Y-1 Site		C/Ann Arbor/1/50 ^2^	C/Yamagata/ 10/89 ^3^
		J9	U9	Q5	J14	K16	U1	U2	YA3	YA5		
Wild-type		128,000	128,000	64,000	256,000	80	16,000	6400	<20	<20	1280	640
Escape mutant	N173I	320	—	—	—	—	—	—	—	—	2560	1280
	N175S	<20	—	—	<20	—	—	—	—	—	1280	320
	S192L	—	160	—	—	—	—	—	1280	1280	2560	1280
	E193K	—	<20	<20	—	<20	—	—	—	—	320	160
	K235R	—	<20	<20	—	—	—	—	—	—	2560	640
	D269N	—	—	—	<20	<20	—	—	—	—	640	160
	R68W	—	—	—	—	<20	—	—	—	—	2560	1280
	A351V	—	—	—	—	<20	—	—	—	—	1280	640
	L164P	—	—	—	—	—	160	<20	320	80	2560	1280
	K198E	—	—	—	—	—	320	20	40	20	1280	640

^1^ The hemagglutination inhibition (HI) titer is expressed as the reciprocal of the highest antibody dilution that completely inhibited hemagglutination. —, a less than 10-fold lower or higher HI titer than that of the wild-type virus. The sets of the mutant virus and monoclonal antibodies (MAbs) that were used for selection are boxed. ^2^ Antigenicity of C/Ann Arbor/1/50 is identical to that of C/Taylor/1233/47, which is a reference strain of the C/Taylor lineage. ^3^ Antigenicity of C/Yamagata/10/89 is identical to that of C/Yamagata/26/81, which is a reference strain of the C/Yamagata lineage.

**Table 2:** Antigenic properties of escape mutants of the C/Yamagata/15/2004 virus in hemagglutination inhibition (HI) assay.

Viruses	Amino Acid Substitution	HI Titer of MAbs ^1^									HI Titer of Chicken Antiserum against:	
		A-1 Site				A-2 Site	A-3 Site		Y-1 Site		C/Ann Arbor/1/50 ^2^	C/Yamagata/10/89 ^3^
		J9	U9	Q5	J14	K16	U1	U2	YA3	YA5		
Wild-type		<20	1280	12,800	32,000	320	12,800	1280	12,800	12,800	320	5120
Escape mutant	∆192–195	—	<20	—	—	<20	—	—	<20	<20	320	2560
	∆198	—	<20	80	—	<20	—	—	<20	<20	80	640

^1^ The hemagglutination inhibition (HI) titer is expressed as the reciprocal of the highest antibody dilution that completely inhibited hemagglutination. —, a less than 10-fold lower or higher HI titer than that of the wild-type virus. The sets of the mutant virus and monoclonal antibodies (MAbs) that were used for selection are boxed. ^2^ Antigenicity of C/Ann Arbor/1/50 is identical to that of C/Taylor/1233/47, which is a reference strain of the C/Taylor lineage. ^3^ Antigenicity of C/Yamagata/10/89 is identical to that of C/Yamagata/26/81, which is a reference strain of the C/Yamagata lineage.

## Data Availability

Data are available in the article and in supplementary materials.

## Supplementary Materials

The following are available online at https://www.mdpi.com/1999-4915/13/3/401/s1: Table S1: Hemagglutination inhibition (HI) titers of antigenic reference strains of influenza C virus. Table S2: Hemagglutination inhibition (HI) titers of representative antigenic mutants among the natural isolates.
