# Supplementary material for: Growth Kinetics of Influenza C Virus Antigenic Mutants That Escaped from Anti-Hemagglutinin Esterase Monoclonal Antibodies and Viral Antigenic Changes Found in Field Isolates
Source: Viruses. 2021 Mar 3;13(3):401. doi: 10.3390/v13030401 (PMC7998938; doi:10.3390/v13030401)
Supplement: Supplementary file 1 [file viruses-13-00401-s001.pdf]

Supplementary Table S1: Hemagglutination inhibition (HI) titers of antigenic reference strains of influenza C virus.

	HI titer														
	MAbs of respective antigenic sites								Chicken antiserum against:						HE lineage
	A-1				A-3		Y-1								
Reference strains	J9	U9	Q5	J14	U1	U2	YA3	YA5	C/Ann Arbor/1/50	C/Aichi/1/81	C/Mississippi/80	C/Yamagata/10/89[a]	C/Kanagawa/1/76	C/Sao Paulo/378/82	
C/Ann Arbor/1/50	64000	64000	16000	256000	16000	6400	<[b]	<	1280[c]	320	320	640	640	640	C/Taylor
C/Aichi/1/81	<	16000	<	256000	640	80	<	<	80	640	320	320	5120	320	C/Aichi
C/Mississippi/80	<	<	<	256000	32000	12800	<	<	160	160	2560	160	160	320	C/Mississippi
C/Yamagata/26/81	<	640	1600	64000	40	160	12800	12800	320	320	320	2560	640	640	C/Yamagata
C/Kanagawa/1/76	<	40	320	128000	<	<	<	<	160	320	160	160	2560	320	C/Kanagawa
C/Sao Paulo/378/82	<	16000	800	64000	32000	12800	12800	6400	640	320	320	320	1280	1280	C/Sao Paulo

[a] The HE antigenicity of C/Yamagata/10/89 is identical to that of C/Yamagata/26/81 (10).
[b] less than 20
[c] HI titers obtained with antisera against the homologous strains are underlined.

Supplementary Table S2: Hemagglutination inhibition (HI) titers of representative antigenic mutants among the natural isolates.

		HI titer													
		MAbs of respective antigenic sites								Chicken antiserum against:					
		A-1				A-3		Y-1							
Viruses	Amino acid change	J9	U9	Q5	J14	U1	U2	YA3	YA5	C/Ann Arbor/1/50	C/Aichi/1/81	C/Mississippi /80	C/Yamagata /10/89[a]	C/Kana gawa/1/76	C/Sao Paulo/378/82
C/Yamagata lineage															
C/Yamagata/26/81	—	<[b]	640	1600	64000	40	160	12800	12800	320	320	320	2560	640	640
C/Yamagata/11/88	D269N	<	80	1600	<	160	<	12800	6400	320	160	160	2560	320	320
C/Miyagi/2/92	E198K	<	640	320	16000	32000	12800	25600	25600	320	320	320	2560	640	640
C/Kanagawa lineage															
C/Kanagawa/1/76	—	<	40	320	128000	<	<	<	<	160	320	160	160	2560	320
C/Miyagi/11/2002	D176N	<	16000	320	256000	<	<	<	<	160	640	320	320	2560	640
C/Miyagi/4/2002	E198K	<	40	80	128000	800	160	80	40	160	640	320	320	2560	640
C/Miyagi/31/2002	D176N, E198K	<	8000	80	128000	640	320	80	40	160	320	320	320	2560	640
C/Yamagata/29/2004	D269N	<	40	80	<	<	<	<	<	80	160	80	160	1280	160
C/Sao Paulo lineage															
C/Sao Paulo/378/82	—	<	16000	800	64000	32000	12800	12800	6400	640	320	320	320	1280	1280
C/Yamagata/30/2014	K190N	1280	64000	800	64000	8000	1600	<	<	320	320	320	320	1280	1280
C/Yamagata/1/2016	D125N, K190N	128000	32000	1600	128000	32000	12800	<	<	320	320	320	320	1280	1280

[a] The HE antigenicity of C/Yamagata/10/89 is identical to that of C/Yamagata/26/81 (10).
[b] less than 20
